# The Nitrites and Their Action upon Hypertension

**Published:** 1909-05-01

**Authors:** 


### THE NITRITES AND THEIR ACTION UPON HYPERTENSION.

The exact extent, duration, dosage, and other points of therapeutic importance in the action of various vaso-dilator drugs in common use have been very carefully studied by Dr. E. Matthew, who has lately published his conclusions in a contemporary.* The author remarks that no extended series of observations upon the blood pressure of patients to whom these drugs are exhibited has been published in which accurate hæmomanometric records have been taken.

It is to be emphasised that, within limits, high blood pressure is not necessarily harmful to the patient in whom it exists; it is primarily a compensatory change, which may or may not achieve results of value to the economy, though, unfortunately, it is found often to tend to be progressive and ultimately harmful. The indications for and against interference with hypertension have not yet been worked out: but when in any given case it seems advisable to attempt by drugs to reduce an abnormally high blood pressure, it will be found best to rest content with a measure of success which still leaves the patient with a higher pressure than is usual in health. That is to say, a reduction of about 30 mm. of mercury gives better results in the relief of symptoms than any greater diminution will do, even when the original pressure is 80 or 100 mm. in excess of the normal maximum.

Again, it is a matter of common experience among physicians who prescribe nitrites or other vaso-dilators that there are great variations in the rapidity with which the different drugs produce an effect, and still greater variations in the length of time during which the effects last. For angina pectoris, asthma, and spasmodic conditions generally, where a rapid and considerable action is required, amyl nitrite is the dilator *par excellence*; its action, though transient, is both immediate and powerful, and its diffusibility insures its efficient absorption. As for the nitrites, which are given in the hope of securing a more prolonged vaso-dilatation, nitro-glycerine is the most rapid. The pharmacopœial preparation of this is liquor trinitrini, and its official dose is one to two minims. The latter quantity produces in most cases of hypertension a fall of pressure which begins always within ninety seconds, generally within a minute. The maximum fall is reached at the end of four and a half minutes, and is about 28 mm. of mercury; the subsequent rise is slower than the fall, but in all cases the effect of the drug passes off within half an hour. Further, nitroglycerine itself, given in tabloid form, has no action whatever of any sort; it is to be supposed that the tabloids are either pharmacologically inert, or that they remain undissolved.

The next most rapid in action are the nitrites of sodium and potassium, whose freshly prepared solutions are given in doses containing two or three grains. The onset of dilatation, judging by the manometric reading, occurs in about five minutes; the maximum fall averages 32 mm., is complete in fourteen minutes, and is maintained for forty or fifty minutes; the natural level is reached about two hours after administration. It is found that such a dose given three times a day will keep the pressure down, not constantly at the lowest level of a single dose, but quite markedly. Tolerance is, apparently, not established, whereas with liquor trinitrini the patient will gradually respond only to larger and larger doses up to 60 minims or more.

Erythrol tetranitrate and mannitol hexanitrate are slower and more persistent still. The former was given in two proprietary forms, one of which (Martindale's) acted quite satisfactorily, whereas the other had not the slightest effect. Erythol tetranitrate in half-grain and one-grain doses begins to act in five and a half minutes, and produces its maximum result in twenty-two minutes; mannitol hexanitrate (one grain) in twelve and a hundred minutes respectively. Both produce about the same degree of fall, namely, 35 mm.; both maintain their full effect for one to two hours, and do not cease to act altogether for five or six hours. There is no tendency to the establishment of tolerance; a dose of half to one grain will secure permanently a fall of about 30 mm. if given thrice daily. These two drugs are not so well borne as the inorganic nitrites; occasionally severe symptoms of over-action, probably due to individual susceptibility, are caused. Lastly, cobalto-nitrite of potassium, which has been employed chiefly in America, was tested. It is apparently quite inert, and has no vaso-dilator action at all.

It is worthy of note that Dr. Matthew finds some patients with marked hypertension who fail to respond to vaso-dilator drugs, or respond but very slightly. There are others in whom even an actual rise of blood pressure is produced; such patients are the subjects of advanced chronic inflammatory nephritis or arteriosclerosis, and in the earlier stages of their disease they respond to medication in the usual way. In cardiac and renal cases in which œdema is marked the nitrites may also prove disappointing; after the œdema has cleared up the usual vaso-dilator action may reappear.

The knowledge gained by this extremely careful and painstaking research places medical practitioners in possession of a much more trustworthy guide to the use of vaso-dilator drugs than they have hitherto had. It demonstrates the considerable advantages of ordering sodium and potassium nitrites instead of liquor trinitrini for persistent high tension, and the precise characters of erythrol and mannitol nitrites, drugs of which all students hear in their pharmacological lectures but few ever see administered. In view of the prevailing view that potassium salts depress the heart more than those of sodium, it is interesting to observe that Dr. Matthew can detect no difference in their reaction upon the blood pressure.

* *The Quarterly Journal of Medicine*. April, 1909.

